# The step further smile virtual planning: milled versus prototyped mock-ups for the evaluation of the designed smile characteristics

**DOI:** 10.1186/s12903-020-01145-z

**Published:** 2020-06-05

**Authors:** Antonino Lo Giudice, Luca Ortensi, Marco Farronato, Alessandra Lucchese, Erica Lo Castro, Gaetano Isola

**Affiliations:** 1 Department of General Surgery and Medical-Surgical Specialties, Section of Orthodontics, School of Dentistry, University of Catania, Policlinico Universitario “Vittorio Emanuele”, Via Santa Sofia 78, 95123 Catania, Italy; 2 Department of General Surgery and Medical-Surgical Specialties, Section of Prosthodontist, School of Dentistry, University of Catania, Policlinico Universitario “Vittorio Emanuele”, Via Santa Sofia 78, Catania, 98123 Italy; 3 Department of Medicine, Surgery and Dentistry, Section of Orthodontics, University of Milan, Milan, Italy; 4 Department of Orthodontics, Vita-Salute San Raffaele University, Milan, Italy; 5 Private Practice, Catania, Italy; 6 Department of General Surgery and Medical-Surgical Specialties, School of Dentistry, University of Catania, Catania, Italy

**Keywords:** Smile virtual planning, Digital dentistry, Smile aesthetics, Prototyped mock-up, Milled mock-up

## Abstract

**Background:**

Mock-up based approach allows the preview of the aesthetic rehabilitation, however, it is crucial that the mock-up does not differ from the expected aesthetic outcomes. With CAD-CAM technologies, it is possible to directly create mock-ups from virtual planned smile project, with greater accuracy and efficiency compared to the conventional moulded mock-ups. In this study, we investigated the trueness of mock-ups obtained with milling and 3D printing technology and a full digital work-flow system.

**Methods:**

Ten adults subjects were included and digital smile design/digital wax-up were performed to enhance the aesthetic of maxillary anterior region. Ten milled mock-ups and 10 prototyped mock-ups were obtained from the original .stl file and a digital analysis of trueness was carried out by superimposing the scanned-milled mock-ups and the scanned-prototyped mock-ups to the digital wax-up, according to the surface-to-surface matching technique. Specific linear measurements were performed to investigate and compare the dimensional characteristics of the physical manufactures, the 3D project and the scanned mock-ups. All data were statistically analyzed. A clinical test was also performed to assess the fitting of the final manufacture.

**Results:**

The prototyped mock-ups showed a significant increment of the transversal measurements (*p* < 0.001) while the milled mock-ups showed a significant increment of all vertical and transversal measurements (*p* < 0.001). The prototyped mock-ups showed good fitting after clinical tests while none of the milled mock-ups showed good adaptation (no fitting or significant clinical compensation required). Deviation analysis from the original 3D project reported a greater matching percentage for the scanned-milled mock-ups (80,31% ± 2.50) compared to the scanned-prototyped mock-ups (69,17% ± 2.64) (*p* < 0.001). This was in contrast with the findings from linear measurements as well as from the clinical test and may have been affected by a reductive algorithmic computation after digitization of physical mock-ups.

**Conclusion:**

Both prototype and milled mock-ups showed a slight dimensional increment comparing to the original 3D project, with milled-mock-ups showing less fitting after clinical tests. Caution must be taken when assessing the trueness of scanned manufacture since an intrinsic error in the system can underestimate the dimensions of the real object.

## Background

Patients’ demand for cosmetic dental treatments is dramatically growing [1, 2]. Among the aesthetic solutions, porcelain veneers (PLV) represents a clinically acceptable, minimally invasive, treatment option to increase smile with the greatest long-term success [3, 4].

Conventional workflows for dental esthetic rehabilitation involves an adequate communication with the dental laboratory technician by using diagnostic waxing and mock-up guide [5–7]. In this respect, it has been demonstrated that tooth preparation is more conservative when a diagnostic mock-up is used compared to the free-hand preparation [8]. Also, diagnostic wax-up enhances the communication with the patient since it shows a realistic preview of the final aesthetic restorations as well as provides clinicians with a better understanding of the patient’s aesthetic expectations [9, 10]. As consequence, patients’ satisfaction with the treatment strictly depends on the consistency of the final product with the mock-up [7, 11].

However, the in-mouth mock-up molding phase is based on complex and operator-dependent procedures. This may lead to low accuracy and inconsistency with patients’ expectations, in particular if the aesthetic result has been previously evaluated and designed in accordance with patients’ needs, as occurring with virtual planning approach [12]. In this respect, virtual planning represents a useful tool to obtain esthetic information for diagnosis and treatment plan as well as for design, fabrication and delivery processes of the definitive restorations [13].

CAD/CAM systems have shown sufficient reliability in the realization of adhesive restorations in aesthetic areas [12–15]; in particular, a recent study [16] demonstrated that milled esthetic mock-ups are much more consistent than those obtained with manual procedure. For instance, the construction of a prototype, based on the virtual assembly, reduces the number of errors in the final product and can represent a fundamental tool for aesthetic rehabilitations and/or prosthetic-driven surgery [16].

To date, the production of CAD-assisted mock-ups can be classified into milling or 3D prototyping, respectively based on material removal and additive process. However, no studies have investigated the accuracy and precision of milled and 3D printed mock-ups produced throughout a full-digital workflow. In fact, previous orthodontic studies assessed only the accuracy of dental models obtained by subtractive manufacturing or additive manufacturing [17, 18] as well as, in prosthetics field, studies were limited to the evaluation of single teeth [19, 20] or partial mouths [21, 22]. Nowadays, with the progresses in 3D imaging, is it possible to comparatively evaluate morphological and dimensional characteristics of anatomical structures or their reproduction. In particular, the surface-to-surface matching technique [23–26] allows the superimposition of 3D objects to evaluate the Euclidean distances between the relative surfaces; also, this digital technique provides, on a 3D color-map, the morphological differences between the superimposed structures in different colors by setting specific levels of tolerance.

Thus, the aim of the present study was to compare the trueness of these two full-digital work-flow for the realization of mock-up for maxillary anterior region. To perform this evaluation, we referred to a specific 3D technology involving digital measurements and the use of surface-to surface matching technique of the two scanned mock-ups. A clinical assessment of fitting of mock-ups was also involved in the study.

## Methods

The study sample consisted of 10 adult subjects (8 females and 2 males, mean age), whose chief compliance was the need of additive restoration in the anterior maxillary area to enhance the smile aesthetic appearance. Subjects were prospectively recruited from a dental private practice in Catania, from June 2019 to December 2019. This study followed the principles laid down by the World Medical Assembly in the Declaration of Helsinki 2008 Helsinki Declaration on medical protocols and ethics and received positive response by the Approval Board of the School of Dentistry, University of Catania (protocol n. 14/19). Inclusion criteria were: adult subjects requiring aesthetic/functional restorations of the maxillary anterior region (canine to canine), good oral hygiene, periodontal health. Exclusion criteria were: missing teeth in the maxillary anterior region, restoration/cavities, history of orthodontic treatment, misalignments and periodontal defects in the maxillary anterior region, severe bruxism or clenching.

### Photographic examination

**Fig. 1:**
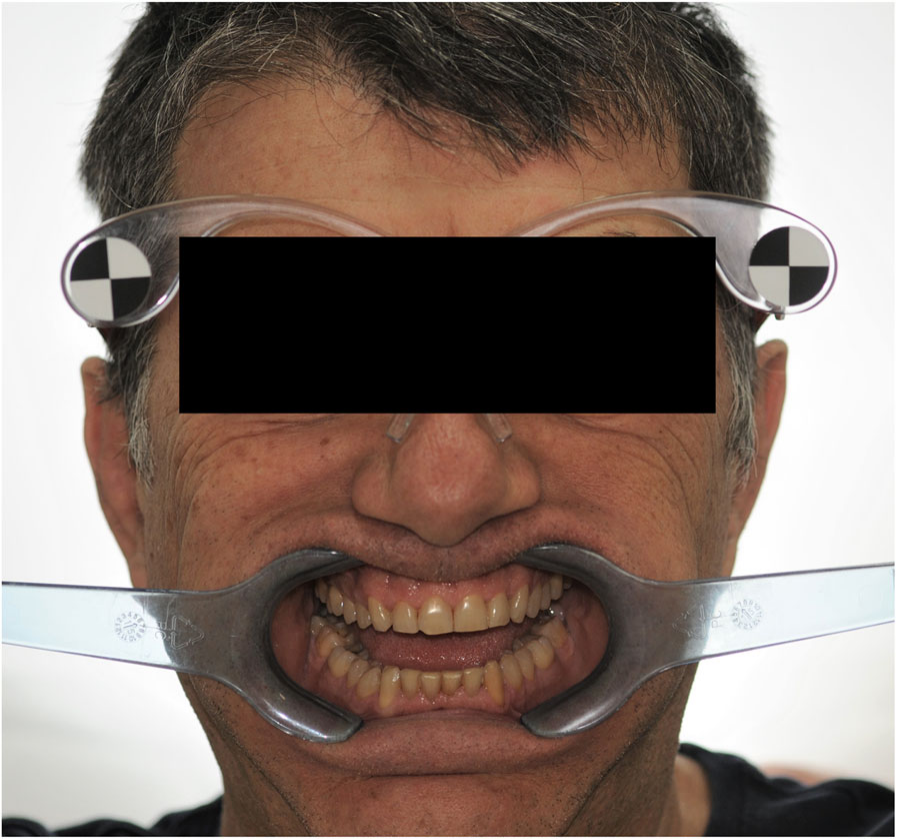
Photograph of the patient’s face with cheek retractors in place. The picture wsd taken with semi-disclosed dental arches, to correctly evaluate the parallelism between the bi-pupillary line and occlusal planes as well as and the congruence between the median and interincisive lines

**Fig. 2:**
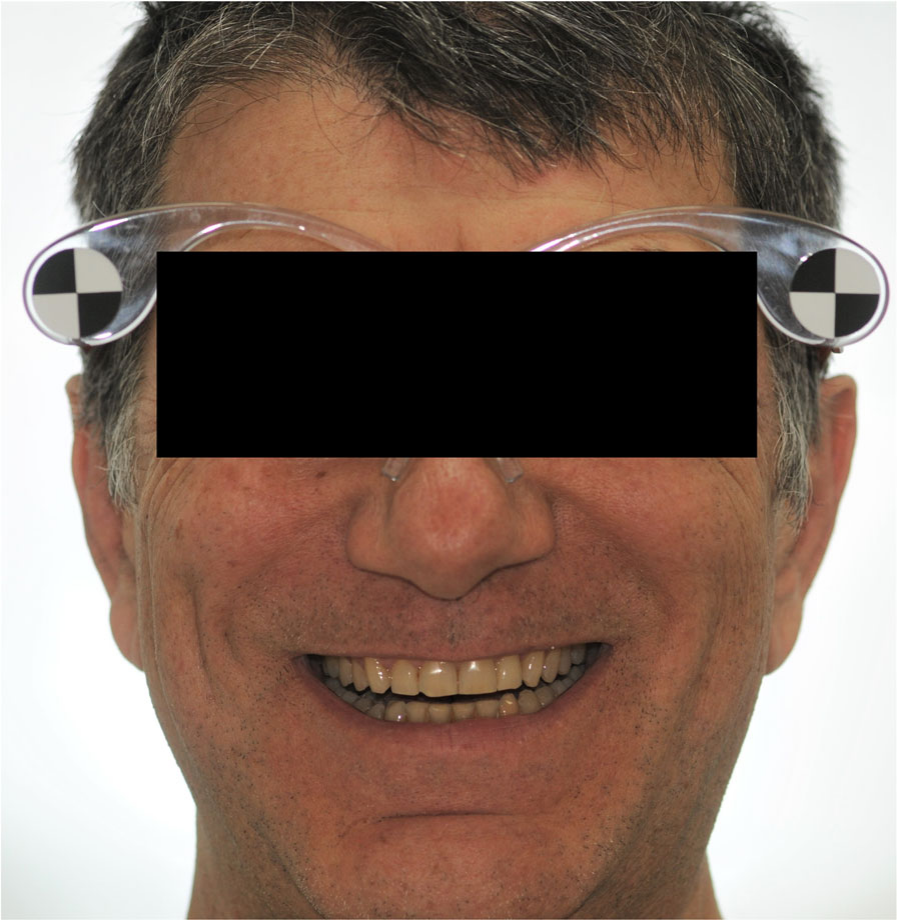
Photograph of the patient’s face without cheek retractors. The picture was obtained by asking patients to smile in order to evaluate the orientation of the incisal plane with respect to the curve of the lower lip, as well as the width of the lateral corridors

After the clinical assessment of smile characteristics (occlusal, phonetic, static and dynamic), each patient underwent digital photographic examination, according to previous documented guidelines of virtual smile design project [26]. In this respect, two full-faces photos of the patient, one with slightly disclosed dental arches and one with a maximum smile, were registered. The first photo (F1) of the face was taken with the retractors, with semi-disclosed dental arches, to correctly evaluate the parallelism between the bi-pupillary line and occlusal planes as well as and the congruence between the median and interincisive lines (Fig. 1). The second photograph (F2) of the face was detected by removing the retractors and asking patients to smile to evaluate the orientation of the incisal plane with respect to the curve of the lower lip, as well as the width of the lateral corridors (Fig. 2) [27].

Standardized photographic records were taken using camera D300 (Nikon Corporation, Minato-ku, Tokyo, Japan) equipped with AF-S VR Micro-Nikkor 105 mm f/2.8G IF-ED macro lens (Nikon Corporation, Minato-ku, Tokyo, Japan) and Metz 15 MS - 1 digital flash system, with LumiQuest pocket bouncer, on a Medical Close-up bracket (CLS Wireless Flash System). Subjects were instructed to be seated behind a line drawn on the floor while the camera was placed at a distance of 1.50 m from the patient and at the same height as the patient's face in a vertical position [28]. Subjects were asked to look at the camera in order to get the bipupillary plane as parallel as possible to the horizontal plane. Subjects were asked to wear specific glasses equipped with an optical measurement system that allowed the clinician to consistently placed the photographic markers over the camera digital grid. Also, the photographic markers provided the conversion of pixels into mm, in order to consistently calibrate the images used during the virtual planning flow. This method increases the reliability of multiple images acquisition as well as the trueness in the subsequent virtual smile design process.

### CAD-CAM workflow

#### Step 1- virtual planning

**Fig. 3:**
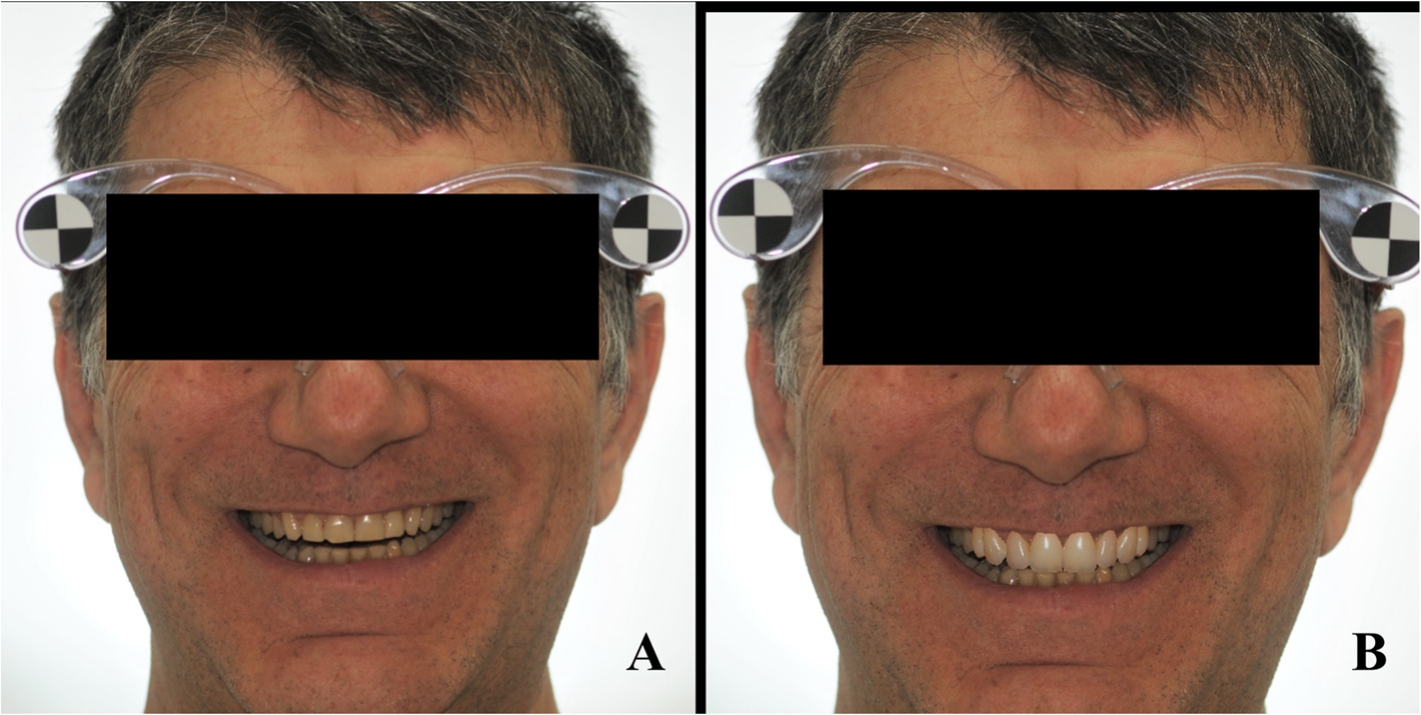
2D smile virtual planning. The virtual project, simulates anterior veneers, was performed and showed to the patients. The procedure was performed by using the 2D DSS system software (version 1.11.1-alpha.1, Digital Smile System Srl, Italy. **a** Patient’s original smile, **b** Simulated anterior veneers

The digital photographs were imported into the 2D DSS system (version 1.11.1-alpha.1, Digital Smile System Srl, Italy) for the realization of the virtual planning of the potential aesthetic rehabilitations of the maxillary anterior region (1.3-2.3), aimed at application of veneers and the digital drawing of the new smile, simulating anterior veneers (1.3-2.3) was performed and shown to the patient. The digital restoration project was then realized (Fig. 3).

#### Step 2- realization of digitally designed mock-ups

**Fig. 4:**
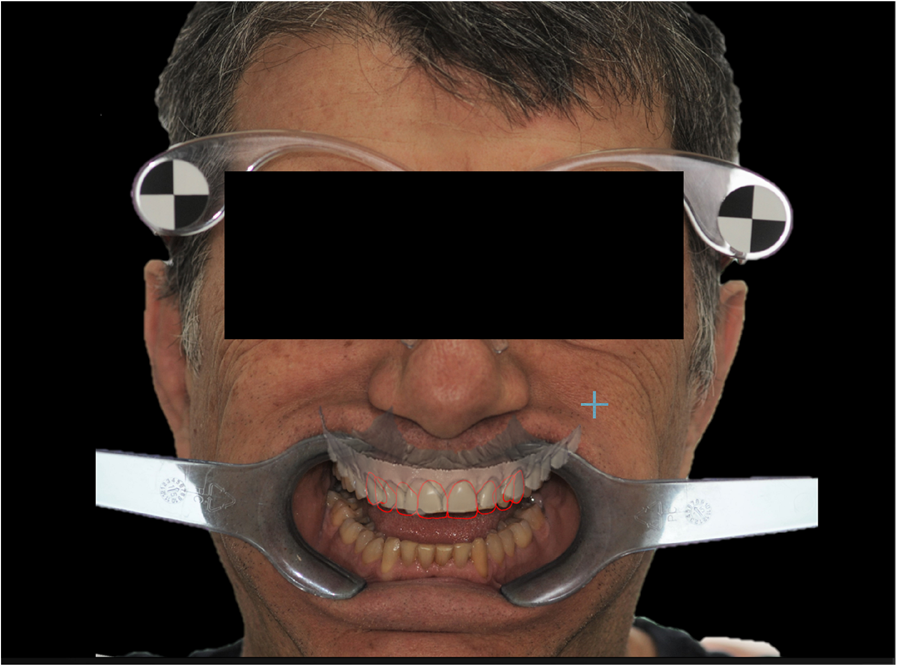
3D smile virtual planning. A three-dimensional digital wax-up using as reference the outlines of the 2D smile design previously performed. The procedure was performed by using the DSS CAD software (DSS3D. Beta.12977, EGS Srl, Italy)

**Fig. 5:**
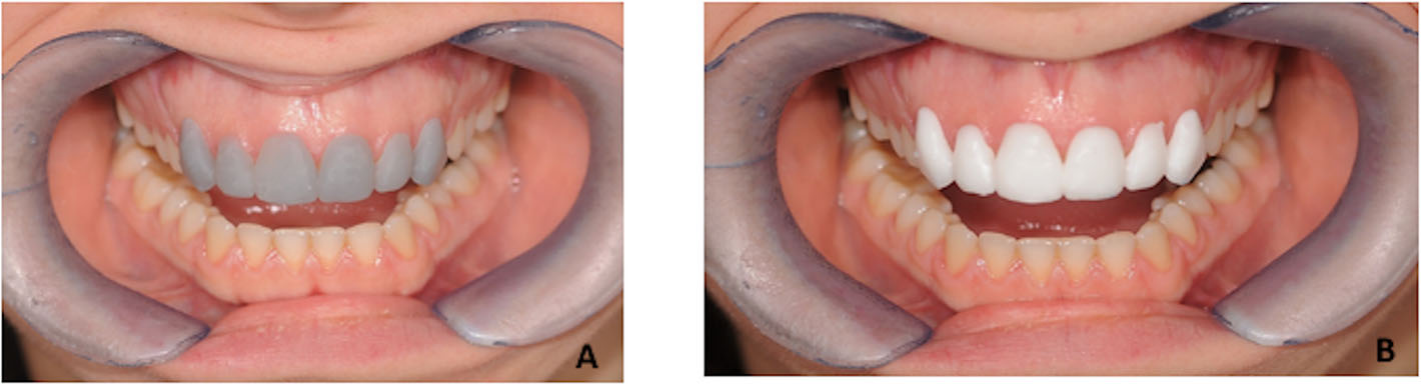
**a** Mockup in methacrylic photoreactive resin (Formlabs, Photopolymer Resin, White (GPWH02)), Formlabs Inc. USA) and **b** in polymethyl methacrylate (Synergy Disk Tempo Multi, Opal, Nobil-Metal SPA, Italy)

In order to obtain a digital wax-up, the stl. files of the patient’s dental arches were registered and align to both F1 and F2 photographs by using the DSS CAD software (DSS3D. Beta.12977, EGS Srl, Italy). This software allows clinicians to design a three-dimensional digital wax-up using as reference the outlines of the 2D smile design previously performed (Fig. 4). The derived .stl file of the digital wax-up was exported and sent to the digital lab for the realization respectively of mock-up (0.4 mm) in methacrylic photoreactive resin (Formlabs, Photopolymer Resin, Gray (GPWH02), Formlabs Inc. USA) and in polymethyl methacrylate (Synergy Disk Tempo Multi, Opal, Nobil-Metal SPA, Italy) (Fig. 5). For the purpose of the present investigation, the 3D printing machine used was the Formlabs form 2 (Formlabs Inc. USA), featuring SLA 3D printing technology. The milling machine used was the CORITEC IMES-ICORE 250i (imes-icore® GmbH, Eiterfeld, Germany) that featured a 5-axis system; the milling sequence of the workpiece involved three progressive internal and external steps of roughing (2 mm), roughing and finishing (1 mm) and finishing (0.6 mm). In order to assure accuracy of the 3D printing, the following procedure were carried out: 1) the liquid resin and the tank were replaced before each print, 2) the digital mock-up was placed in the midst of the printing plate in order to avoid ovalization of the laser beam, 3) the mock-up was positioned with an inclination between 20° and 40° in order to avoid the deformation of the object under its own weight.

#### Step 3- surface-to-surface analysis of milled and prototyped mock-ups

Both milled and prototyped mock-ups were scanned by using optical scanner with structured light technology (SinergiaScan, Nobil-Metal S.p.A, Italy) and the generated .stl files were imported in Exocad software (DentalCad 2.3 Matera, exocad GmbH, Darmstadt, Germania) along with the .stl file of the 3D digital wax-up project. The scan of each prototyped and milled mock-up were registered on this file and the surface-to-surface matching technique was applied to assess the level of trueness of both mock-ups relative to the digital wax-up performed according to the virtual planning.

#### Step 4- clinical test

In the fourth phase of the protocol, the mock-ups were tested in the participants' oral cavity. At this stage, each patient was subjected to occlusal evaluation to discriminate the prosthetic fitting of both methacrylic photoreactive resin and polymethyl methacrylate mock-ups.

Moreover, specific linear measurements were performed to assess potential dimensional alteration in both milled and prototyped mock-ups throughout each stage of the entire CAD-CAM workflow:

1. Upper right central incisor height (rU1h) = measurement taken from the center of incisal margin to the most cranial point of gingival contour of the upper right central incisor
2. Left right central incisor height (lU1h) = measurement taken from the center of incisal margin to the most cranial point of gingival contour of the upper right central incisor
3. Upper right central incisor width (rU1w) = mesio-distal diameter of upper right central incisor meaured at the equator level
4. Left right central incisor width (lU1w) = mesio-distal diameter of upper left central incisor measured at the equator level
5. Canine-to-canine width (CCw) = mesio-distal diameter of anterior frontal group measured at the equator level from the distal margin of upper right canine to the distal margin of upper left canine.

In particular, the reported measurements were performed on:

- 2D digital smile design, by referring to a specific digital caliper in 2D DSS software (Digital Smile System Srl, Italia)
- 3D digital smile project, by using linear measurements function in Exocad.
- scanned MRP and PMMA mock-ups, by using linear measurements function in Exocad.
- MRP and PMMA mock-ups, by using digital caliper (Digital Caliper 0–150 mm, Mitutoyo, Japan).

### Statistical analysis

All the measurements were recorded on Microsoft Excel® spreadsheet (Microsoft, Redmond, WA, USA) and analyzed using SPSS® version 24 Statistics software (IBM Corporation, 1 New Orchard Road, Armonk, New York, USA) with *P* values of less than 0.05 considered statistically significant. The Kolmogorov–Smirnov test and Levene’s test were used to assess respectively the normal distribution and the equality of variance of the data recorded. Since data showed normal distribution (*p* > 0.05) and equality of variance (*p* >0.05), parametric tests were used to evaluate potentially significant differences between data measurements.

The trueness of both prototyped and milled mock-ups was assessed by using the Paired Student’s t test which compared the percentage of matching of scanned-prototyped and scanned-milled mock-ups with the digital 3D project, according to the surface-to-surface analysis.

The two-way analysis of variance (ANOVA) was used to assess if there were statistical differences among the linear measurements obtained at each stage of the entire CAD-CAM workflow. In particular, each linear measurement (rU1h, lU1h, rU1w, lU1w and CCw) obtained from 1) the original 3D project, prototyped and scanned-prototyped mock-ups, 2) the original 3D project, milled and scanned-milled mock-ups were compared and post-hoc comparison tests were performed to assess crossed differences.

## Results

**Fig. 6:**
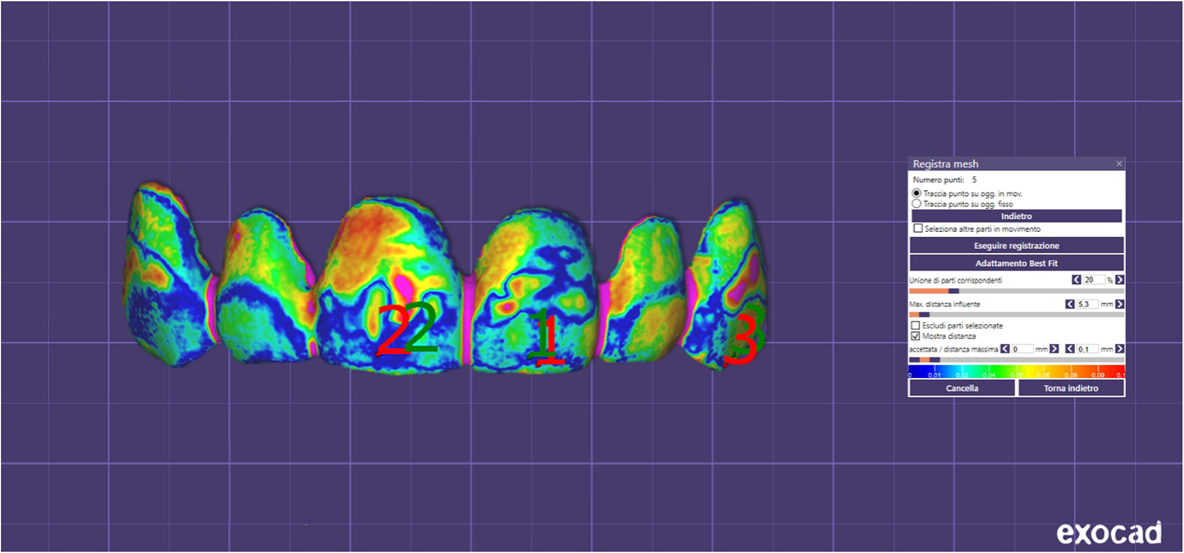
Superimposition of milled mock-ups with the 3D project. Color-coded map according to the surface-to-surface analysis

**Fig. 7:**
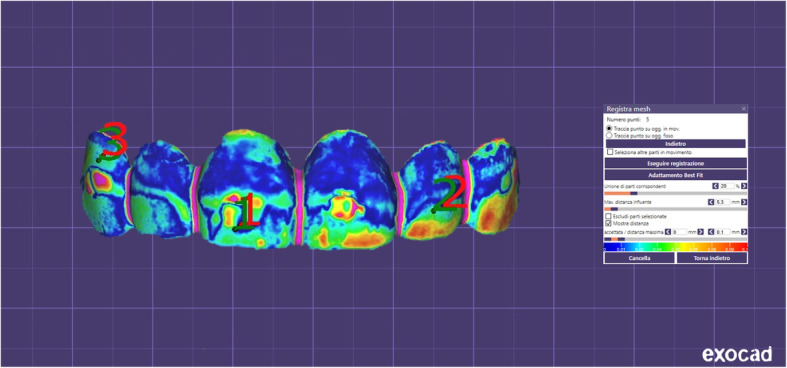
Superimposition of prototyped mock-ups with the 3D project. Color-coded map according to the surface-to-surface analysis

Table 1 shows inferential statistics of deviation analysis of milled and prototyped mock-ups relative to the 3D project. The surface-to-surface analysis showed a significant higher percentage of matching between the 3D project and milled mock-ups (75,31 %) than between the 3D project and the prototyped mock-ups (63,17 %) (*p* < 0.001), according to the paired Student’s t test. Figures 6 and 7 show respectively the color-coded map of milled and prototyped mock-ups.

**Table 1:** Matching percentage of prototyped and milled mock-ups with 3D project, according to deviation analysis

	Total	% Matching^**a**^	SD	Significance
**3D Project/Prototyped**	10	69.17	2.64	*p* < 0.001
**3D Project/Milled**	10	80.31	2.50

Tables 2 and 3 show inferential statistics respectively for the linear measurements recorded throughout the digital work-flow for the production of prototyped and milled mock-ups. According to the ANOVA analysis, significant differences were found among 3D project, prototyped and prototyped-scanned anterior mock-ups (*p* < 0.001) (Table 1) and among 3D project milled and milled-scanned anterior mock-ups (*p* < 0.001) (Table 2) for each linear measurement assessed.

**Table 2:** Comparison of linear measurements (mm^3^) performed on 3D project, prototyped and prototyped-scanned anterior mock-ups

	Total sample	Upper right central incisor				Upper left central incisor				Total Diameter	
		rU1h	Significance	rU1w	Significance	lU1h	Significance	lU1w	Significance	CCw	Significance
**3D Project [a]**	10	10,72 ± 0,52 [c]	*p* < 0.001	8,63 ± 0,40 [b,c]	*p* < 0.001	10,68 ± 0,44 [c]	*p* < 0.001	8,60 ± 0,46 [b]	*p* < 0.001	39,43 ± 2,57	*p* < 0.001
**Prototyped [b]**	10	10,74 ± 0,48 [c]		8,87 ± 0,30 [a,c]		10,66 ± 0,52 [c]		8,85 ± 0,55 [a,c]		39,92 ± 3,37	
**Prototyped - scanned [c]**	10	10,47 ± 0,38 [a,b]		8,69 ± 0,21 [a,b]		10,26 ± 0,48 [a,b]		8,62 ± 0,69 [b]		39,39 ± 3,01	

*P* values set at *p* < 0.05 and based on Two-ways analysis of Variance (ANOVA)

*rU1h* right upper central incisor height, *rU1w* right upper central incisor width, *lU1h* left upper central incisor height, *lU1w* left upper central incisor width, *CCw* canine to canine width

**Table 3:** Comparison of linear measurements (mm^3^) performed on 3D project, milled and milled scanned anterior mock-ups

	Total sample	Upper right central incisor			Significance	Upper left central incisor			Significance	Total Diameter	Significance
		rU1h	Significance	rU1w	Significance	lU1h	Significance	lU1w	Significance	CCw	Significance
**3D Project**	10	10,72 ± 0,52 [b]	*p* < 0.001	8,63 ± 0,40 [b]	*p* < 0.001	10,68 ± 0,44 [b]	*p* < 0.001	8,60 ± 0,46 [b]	*p* < 0.001	39,43 ± 2,57 [b,c]	*p* < 0.001
**Milled**	10	11,1 ± 0,39 [a,c]		8,90 ± 0,22 [a,c]		10,97 ± 0.49 [a,c]		8,92 ± 0.48 [a,c]		40,29 ± 2.72 [a,c]	
**Milled - scanned**	10	10,70 ± 0,44 [b]		8,59 ± 0,36 [b]		10,69 ± 0,37 [b]		8,58 ± 0,59 [b]		39,33 ± 2,43 [a,b]	

*P* values set at *p* < 0.05 and based on Two-ways analysis of Variance (ANOVA)

*rU1h* right upper central incisor height, *rU1w* right upper central incisor width, *lU1h* left upper central incisor height, *lU1w* left upper central incisor width, *CCw* canine to canine width

According to the post-hoc tests, the prototyped mock-ups showed a significant increment of transversal linear measurements (rU1w, lU1w, CCw) (*p* < 0.001) compared to the 3D project while the scanned-prototyped mock-ups showed a significant reduction of all vertical and transversal linear measurements (rU1w, lU1w, CCw, rU1h, lU1h) compared to the prototyped mock-ups (*p* < 0.001). No differences were found in vertical linear measurements between the original 3D project and prototyped mock-ups (*p* > 0.05) (Table 1).

The milled mock-ups showed a significant increment of all vertical and transversal linear measurements (rU1w, lU1w, CCw, rU1h, lU1h) (*p* < 0.001) compared to the 3D project while the scanned-milled mock-ups showed a significant reduction of all vertical and transversal linear measurements (rU1w, lU1w, CCw, rU1h, lU1h) compared to the prototyped mock-ups (*p* < 0.001). No significant differences were found between the scanned-milled mock-ups and the original 3D project except for the CCw measuerement where a slightly significant reduction was found (*p* > 0.05) (Table 2).

Finally, the clinical investigation of mock-ups fitting showed (data not showed) good engagement for the prototyped mock-ups (no changes or adaptations required) and poor engagement for the milled mock-ups (no fitting or significant clinical adaptations required).

## Discussion

In contemporary dentistry, any efforts should be made to enhance the communication of diagnostic and therapeutic information to the patients and between dental specialists. In this respect, virtual planning along with mock-up based approach increases the predictability of the aesthetic restorations since this work-flow improves the understanding of the patients’ expectations as well as enhance the information-sharing process between prosthodontists and lab technicians [5, 29]. Thus, it is possible to correlate the wax-up to the patient’s facial and smile characteristics, reducing the risk of discordance between the wax-up and the clinically tested mock-up [30–32]. By using virtual planning, however, it is crucial that the mock-up does not differ from the results pre-visualized in the software, in order to avoid communication problems and loss of patient’s confidence.

Before explaining the data of the trueness of the mock-ups, a brief comment of the protocol presented in this study for the aesthetic virtual planning is mandatory. It could be argued that using a software including both 2D and 3D functionalities (for example, Exocad) would expedite the work-flow making the entire process more fluent and efficient. Instead, we preliminary used a 2D software for smile design for the following reasons: 1) the method applied for standardization and calibration of the images (eyewear) cannot be used with Exocad, 2) it allows clinicians to easily and efficiently drawing the new smile outlines as well as modifying the virtual planning with the patient instantly seeing the changes and the final outcomes according to his/her concerns. In this respect, the virtual planning should be handled exclusively by the clinician and should not be delegated to the technicians.

Mock-up molding phase is a complex process with low reliability in specific procedures such as the positioning of the matrix, the pressuring of silicon key during resin hardening and the resin removal [33]. A recent well-conducted study [16] found significant differences in the accuracy between moulded and milled mock-ups (full digital work-flow) compared to their original wax-up. For instance, authors [16] concluded that the use of moulded mock-ups would reduce the accuracy of the previewing of the final aesthetic result and that the full digital wax up with milling technology is more reliable for the same purpose. To the best of our knowledge, this is the first study in literature investigating the trueness of two different mock-ups both produced with a full digital work-flow technology, respectively the milled mock-up (methacrylic photoreactive resin) and the prototyped mock-up (polymethyl methacrylate). The digital project of the final mock-ups has been realized following the guidelines of 2D/3D digital smile design and by using dedicate software [12, 34].

Compared to the original digital 3D project, the prototyped mock-ups showed a significant increment of the transversal measurements (rU1w: + 0,24 mm^3^, lU1w: + 0,25, mm^3^, CCw: + 0,49 mm^3^) while the milled mock-ups showed a significant increment of all vertical (rU1h: + 0,38 mm^3^, lU1h: + 0,29 mm^3^) and transversal measurements measurements (rU1w: + 0,27 + mm^3^, lU1w: + 0,32, CCw: + 0,86 mm^3^). Such dimensional differences with the original 3D project were clinically negligible for the prototyped mock-ups, if we consider that they showed a good fitting after clinical tests (data not shown). Conversely, none of milled mock-ups produced in this study reported good clinical fitting (i.e., no stable engagement or significant clinical adaptations required) (data not shown) and none was used for subsequent phonetic and occlusal clinical tests. For assumption, the production of thin objects (mock-ups or veneers) by using milling machine may present some difficulties since the bur (cutting tool) may not adequately penetrate the resin block thus, increasing the final dimension of the object. In this regard, we are conscious that our study provides some new evidence as well as new unanswered questions and further studies are certainly required.

Moreover, the total diameter (CCw) showed the maximum deviation range compared to the other measurements in both milled and prototyped mock-ups, this could be attributed to the differences in the curvature of the arch in the canine region [35, 36]. In this respect, caution must be taken when analyze this linear parameter for aesthetic rehabilitation purpose, in particular when standard 3D virtual templates are selected from the digital library available within digital smile design software.

Before performing the clinical tests, both milled and prototyped mock-ups were scanned and the obtained .stl files were superimposed to the original 3D project in order to assess the trueness of the final products. Also, the same linear measurements were assessed on the digitalized mock-ups and compared to those performed on physical mock-ups and on the 3D project. According to the deviation analysis, we found that the scanned-milled mock-ups showed greater trueness compared to the scanned-prototyped mock-ups, as confirmed by the differences in the percentage of matching with the 3D digital project (3D Project/Milled matching = 80,31 %; 3D Project/Prototyped matching = 69,17 %). These data are in contrast with the findings obtained from the clinical tests as well as with the measurements performed on physical mock-ups, however, they can be explained if we consider the general trend of the linear measurements performed on the scanned mock-ups. In fact, we found a significant reduction of all linear parameters in both scanned-milled and scanned-prototyped mock-ups compared to the respective physical mock-ups. This is in agreement with previous studies suggesting that after CAD/CAM digitization the same measurements performed on virtual environment can be reduced [37], probably due to reductive algorithmic computation.

Consequently, the dimensional increment registered in both physical milled and prototyped mock-ups were, somehow, counterweighed in the virtual environment. In particular, the scanned-milled mock-ups showed no dimensional differences compared to 3D project, while the scanned-prototyped mockups showed a statistically significant reduction in both vertical and transversal measurements evaluated. Again, this is in contrast with the linear measurements performed on the physical mock-ups, and would explain why the scanned-milled mock-ups showed greater trueness compared to the scanned-prototyped mock-ups, according to the deviation analysis. In the light of these findings, caution should be taken when testing the trueness of scanned mock-ups or veneers since results obtained in virtual environment from digitized objects (mock-ups in this case) may not directly reflect the clinical validation of the prosthetic rehabilitation.

We used the gray resin for the production of prototyped mock-ups. This choice was taken for the purpose of the present research (the assessment of trueness of the mock-ups) as well as for facilitating the examination of fitting. From the functional perspective, the clinical test for validating the adaptability of the 3D printed mock-ups should be performed using an opaque resin in order to facilitating the detection of areas of premature contacts of poor fitting. This also enhances the communication between clinicians and lab technicians. From the aesthetical perspective instead, the opaque resin is not adequate to show to the patients the realistic preview of the final aesthetic restorations. In this respect, communication with the patient should be performed one step before the functional clinical test, by the digital preview of the designed smile or by in-mouth visualization of a white mock-up produced for this purpose.

Last but not least, it must be underlined that the creation of milled or printed mock- ups is suggested in those cases in which significant addition of material is required for functional and aesthetics rehabilitation, otherwise the molded mock-up obtained from the printed model should be still considered the gold standard.

### Limitations

The main advantages of 3D printing over milling machine for the production of prosthesis manufacture are the minimum amount of material required as well as the ability to create multiple products at the same time, increasing clinical efficiency [38, 39]. According to our findings, prototyped mock-ups showed less dimensional changes from the original 3D project compared to the milled mock-ups as well as a better clinical adaptation. However, the present study was based on a small sample size and on a single milled machine and 3D printer, thus our findings should be taken with some caution and definitive conclusion cannot be drawn. In this respect, further ex-vivo/in-vivo studies with large sample size and different milling and prototyping technologies are still required.

## Conclusion

- Both prototype and milled mock-ups showed a slight dimensional increment comparing to the original 3D project.
- These changes were greater for the milled-mock-ups that showed poor fitting in patients’ mouth
  Caution must be taken when assessing the trueness of scanned manufacts since an intrinsic error in algorithm computation can underestimate the dimensions of the real object

## Data Availability

The datasets used and/or analyzed during the current study are available from the corresponding author on reasonable request.

## Abbreviations

DSD: Digital smile design
CAD/CAM: Computer-aided design/computer-aided manufacturing
rU1h: Upper right central incisor height
lU1h: Left right central incisor height
rU1w: Upper right central incisor width
lU1w: Left right central incisor width
CCw: Canine-to-canine width
ANOVA: Two-way analysis of variance
